# Curated incidence of lysosomal storage diseases from the Taiwan Biobank

**DOI:** 10.1038/s41525-023-00372-x

**Published:** 2023-09-23

**Authors:** Meng-Ju Melody Tsai, Miao-Zi Hung, Yi-Lin Lin, Ni-Chung Lee, Yin-Hsiu Chien, Wuh-Liang Hwu

**Affiliations:** 1 Department of Pediatrics, National Taiwan University Hospital and College of Medicine, National Taiwan University, Taipei, Taiwan; 2 Department of Pediatrics, National Taiwan University Hospital Yunlin Branch, Yunlin, Taiwan; 3 Department of Medical Genetics, National Taiwan University Hospital, Taipei, Taiwan; 4 Center for Precision Medicine, China Medical University Hospital, Taichung, Taiwan

**Keywords:** Genetic databases, Disease genetics

## Abstract

Lysosomal storage diseases (LSDs) are a group of metabolic disorders resulting from a deficiency in one of the lysosomal hydrolases. Most LSDs are inherited in an autosomal or X-linked recessive manner. As LSDs are rare, their true incidence in Taiwan remains unknown. In this study, we used high-coverage whole-genome sequencing data from 1,495 Taiwanese individuals obtained from the Taiwan Biobank. We found 3826 variants in 71 genes responsible for autosomal recessive LSDs. We first excluded benign variants by allele frequency and other criteria. As a result, 270 variants were considered disease-causing. We curated these variants using published guidelines from the American College of Medical Genetics and Genomics (ACMG). Our results revealed a combined incidence rate of 13 per 100,000 (conservative estimation by pathologic and likely pathogenic variants; 95% CI 6.92-22.23) to 94 per 100,000 (extended estimation by the inclusion of variants of unknown significance; 95% CI 75.96–115.03) among 71 autosomal recessive disease-associated genes. The conservative estimations were similar to those in published clinical data. No disease-causing mutations were found for 18 other diseases; thus, these diseases are likely extremely rare in Taiwan. The study results are important for designing screening and treatment methods for LSDs in Taiwan and demonstrate the importance of mutation curation to avoid overestimating disease incidences from genomic data.

## Introduction

Lysosomal storage diseases (LSDs) are a group of genetic disorders resulting from a deficiency in one of the lysosomal hydrolases^1^. Most LSDs are inherited in an autosomal (most common) or X-linked recessive (mucopolysaccharidosis type II and Fabry disease) manner^1^. Reported epidemiological data for LSDs vary across countries. In one review article, the combined birth prevalence of LSDs was reported to range from 7.5 per 100,000 live births in British Columbia to 23.5 per 100,000 in the United Arab Emirates (UAE)^2^. The overall birth prevalence of 29 different LSDs studied in the Portuguese population was calculated as 25 per 100,000 live births^3^. The incidence of mucopolysaccharide (MPS) in the United States has been reported as 0.98 per 100,000 live births, with a prevalence of 2.67 per 1 million^4^. The distribution and demographic characteristics of subtypes of LSDs also vary across countries, and one report from Eastern China indicated MPS represented 50.5% of all LSDs^5^. The true incidence of LSDs is unknown in Taiwan due to their rarity.

Understanding the incidence of rare diseases is critical for designing screening and treatment methods. The clinical diagnosis of rare diseases is often delayed, and patients miss the opportunity to receive treatment. Clinical variants can be missed entirely in the diagnostic process. Therefore, screening methods, such as newborn and high-risk screening, are often considered for rare diseases. Newborn screening allows early diagnosis and treatment, even in the presymptomatic period^6^, as shown in the screening of Pompe disease (glycogen storage disease II) and spinal muscular atrophy in Taiwan^7,8^. However, the design of screening approaches can be challenging when the estimates of disease incidences are incorrect. New treatments for rare diseases have recently emerged. These treatments are often expensive, and insurance companies or public health agencies must estimate the treatment cost before making it available, which is impossible without accurate incidence data^9^.

Next-generation sequencing (NGS) has generated a large amount of genomic data from patients and normal populations. These data provide an excellent opportunity to directly estimate disease incidences^10^. For rare diseases, the number of patients with genomic data may be insufficient to accurately estimate disease incidence. Fortunately, regarding recessive diseases, the incidence of carriers is much than that of affected individuals. Thus, disease incidence can be calculated from the carrier rate. However, the disease incidence is often overestimated in studies employing population genomic data due to the inclusion of benign or unknown variants as disease-causing^11^. In this study, we explored the incidence of LSDs using carrier data obtained from the Taiwan Biobank (TWB). We curated all possible disease-causing variants and made a reasonable estimation of the incidence of LSDs in Taiwan.

## Results

### The overall curative incidence of autosomal recessive LSDs

**Fig. 1:**
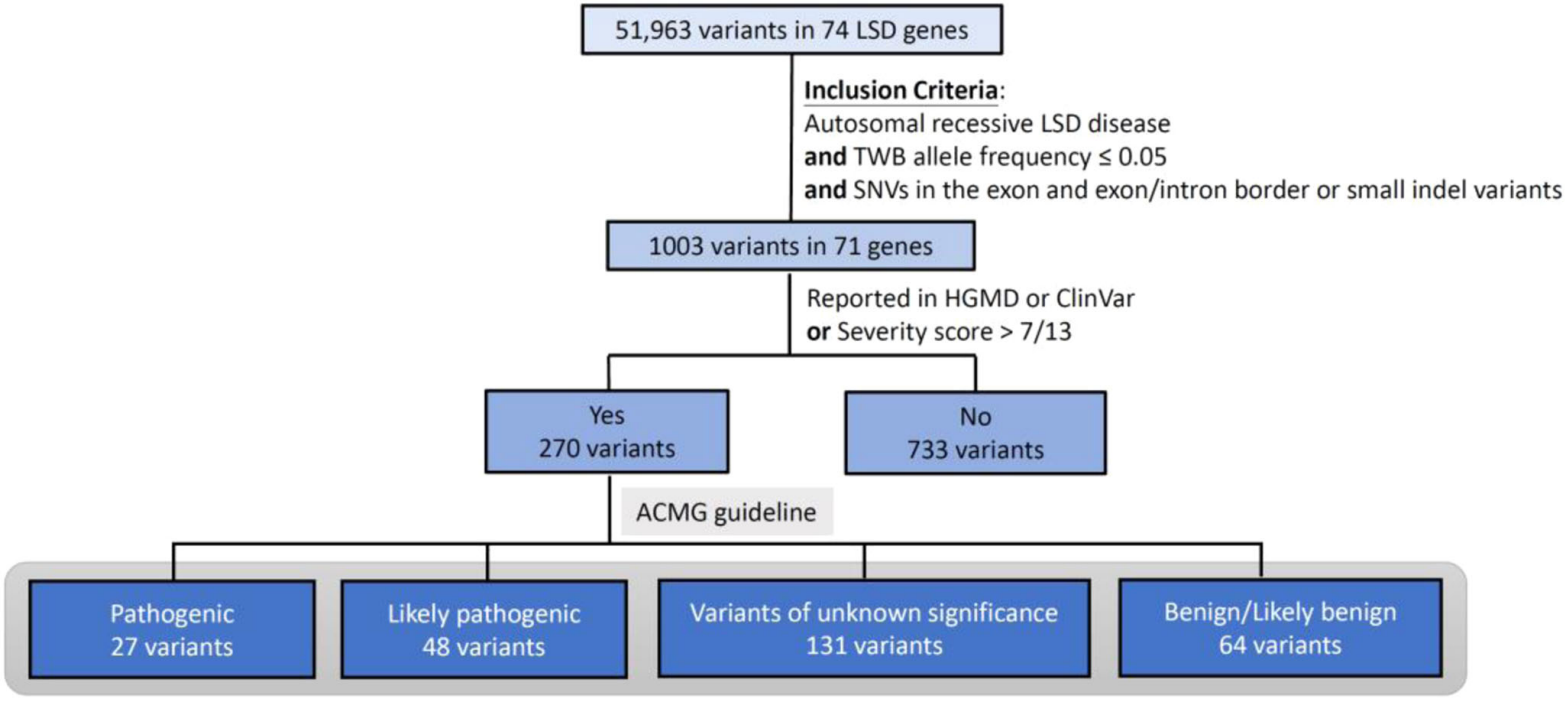
flowchart of this study. Total 51,963 variants in 74 genes related to LSDs were identified from the TWB. We included the 71 genes encoding for the autosomal recessive LSD and included variants with allele frequency <0.05 located in or near the exon region. We included the 71 genes encoding for the autosomal recessive LSD and included variants with allele frequency <0.05 located in or near the exon region; 1003 variants remained for the subsequent analyses. A total of 270 variants in 53 genes were reported in ClinVar or the HGMD as “pathogenic” or or unreported but with a high predicted severity score. We then curated the pathogenicity of these 270 variants according to the 2015 ACMG criteria. LSD lysosomal storage disease, TWB Taiwan Biobank, SNV single nucleotide variant, HGMD Human Gene Mutation Database, ACMG American College of Medical Genetics.

**Fig. 2:**
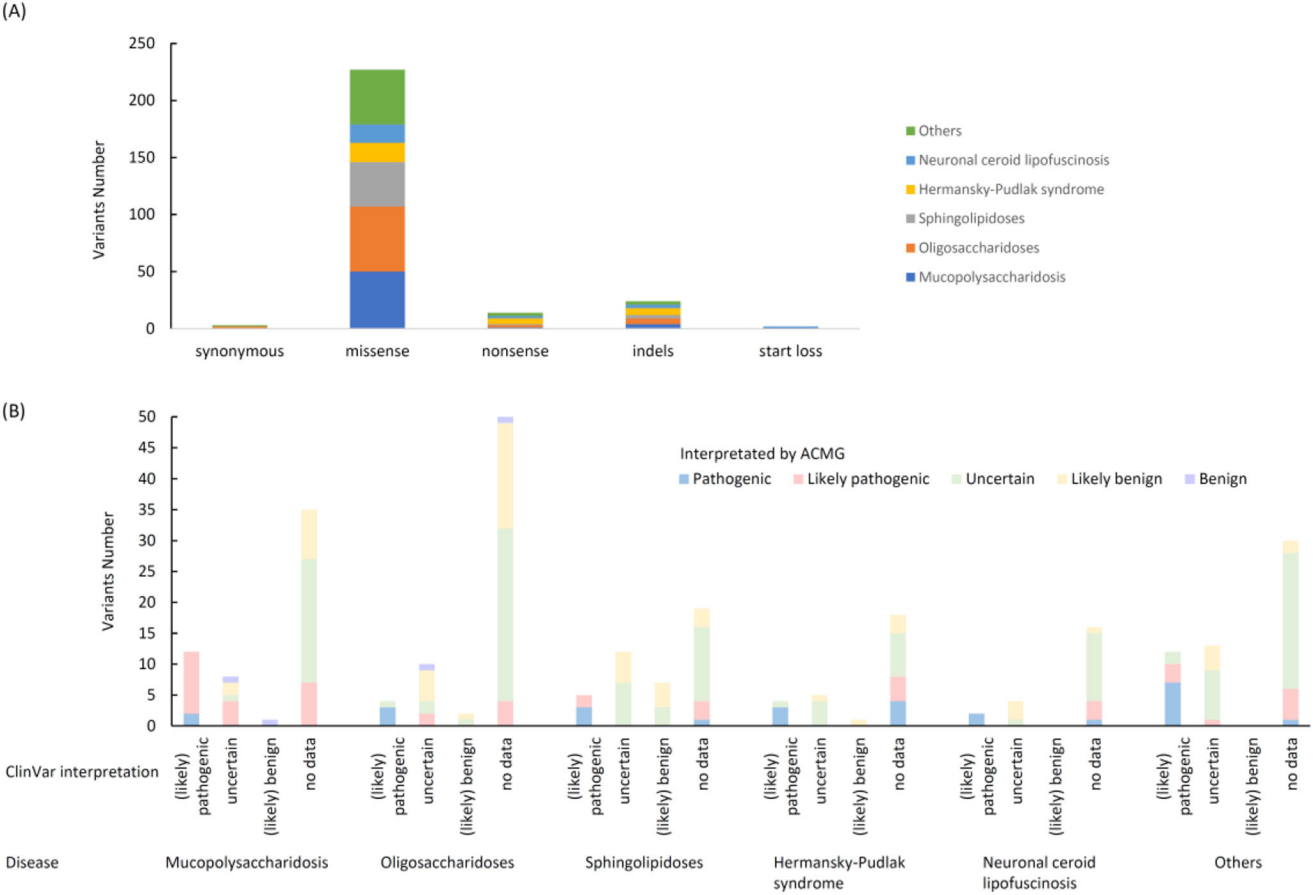
Interpretation of 270 LSD variants. **A** Types of mutations in these 270 variants. **B** Summary of curated ACMG interpretation. The *X*-axis shows the pathogenicity interpretation by ClinVar, and the color shows the pathogenicity interpretation by ACMG (blue: pathogenic, pink: likely pathogenic, green: uncertain, yellow: likely benign, and purple: benign).

**Fig. 3:**
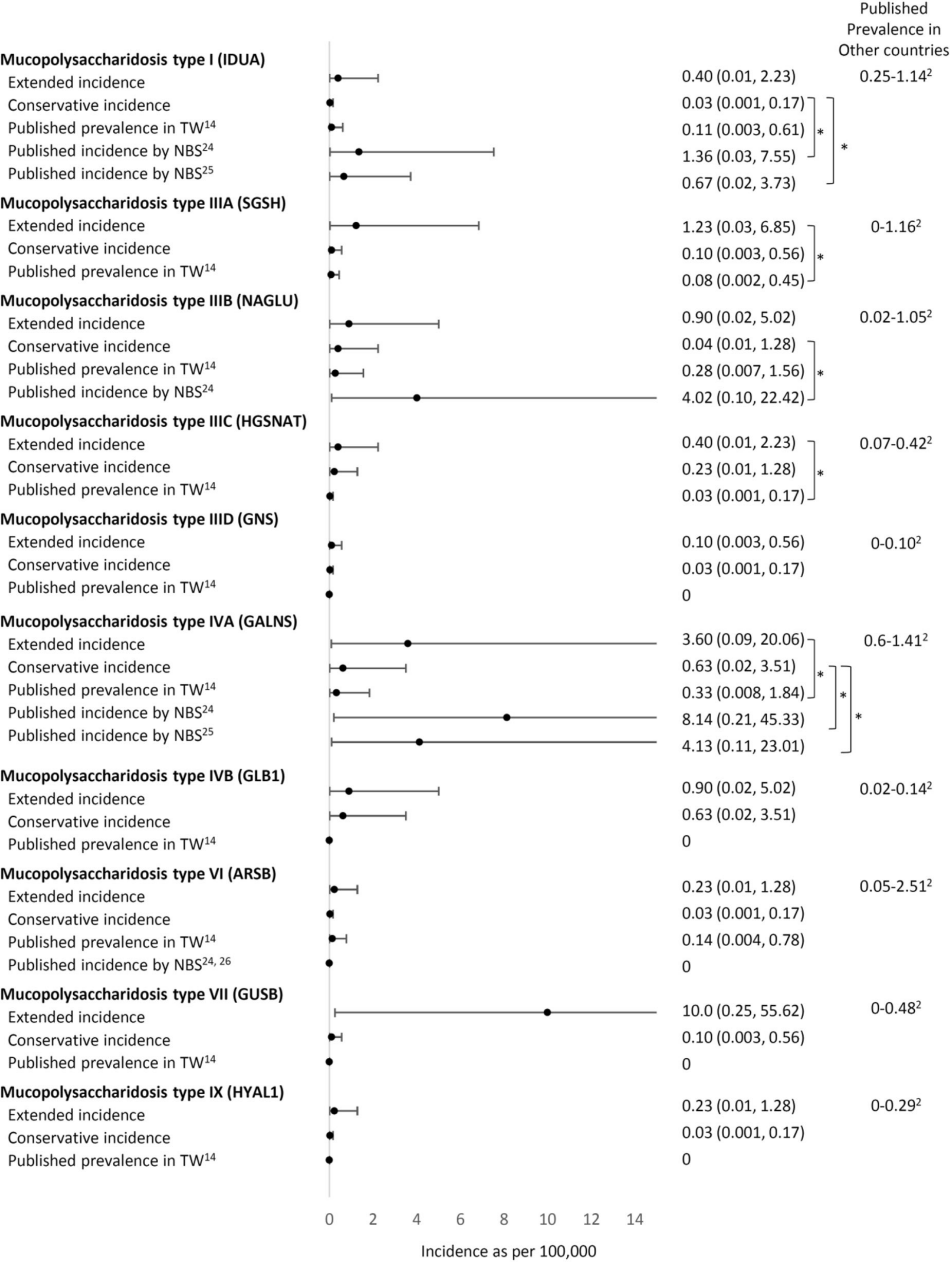

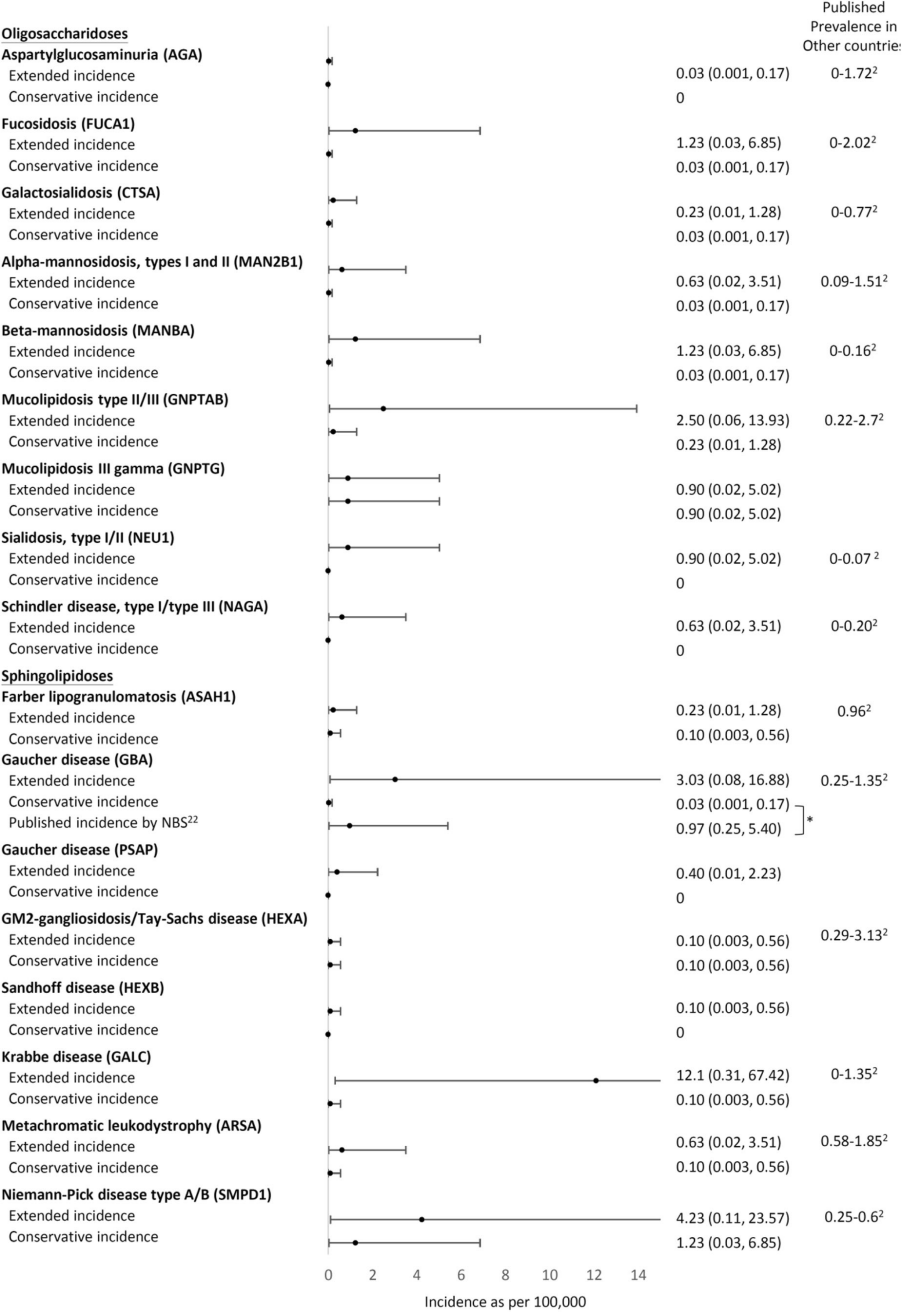

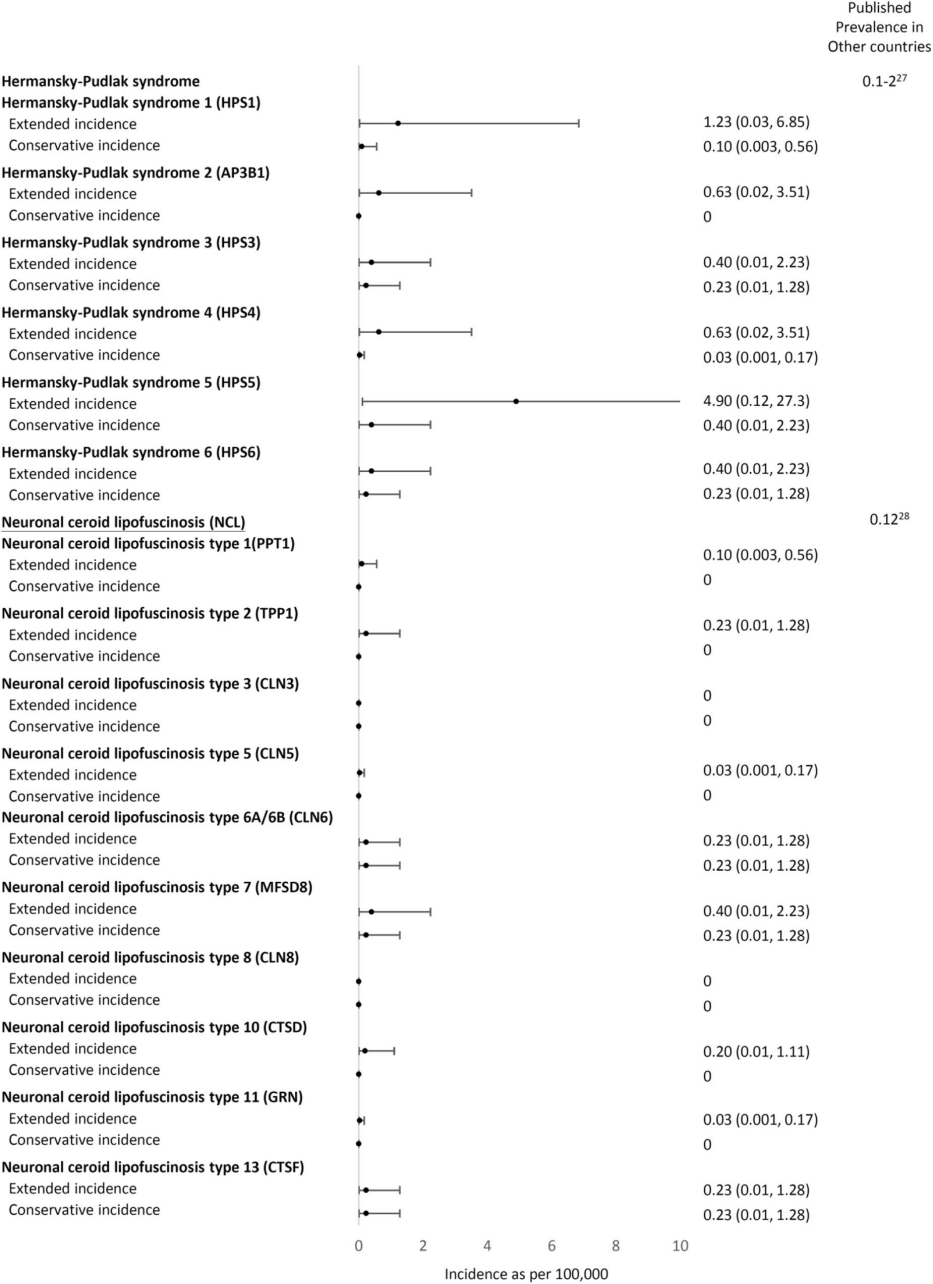

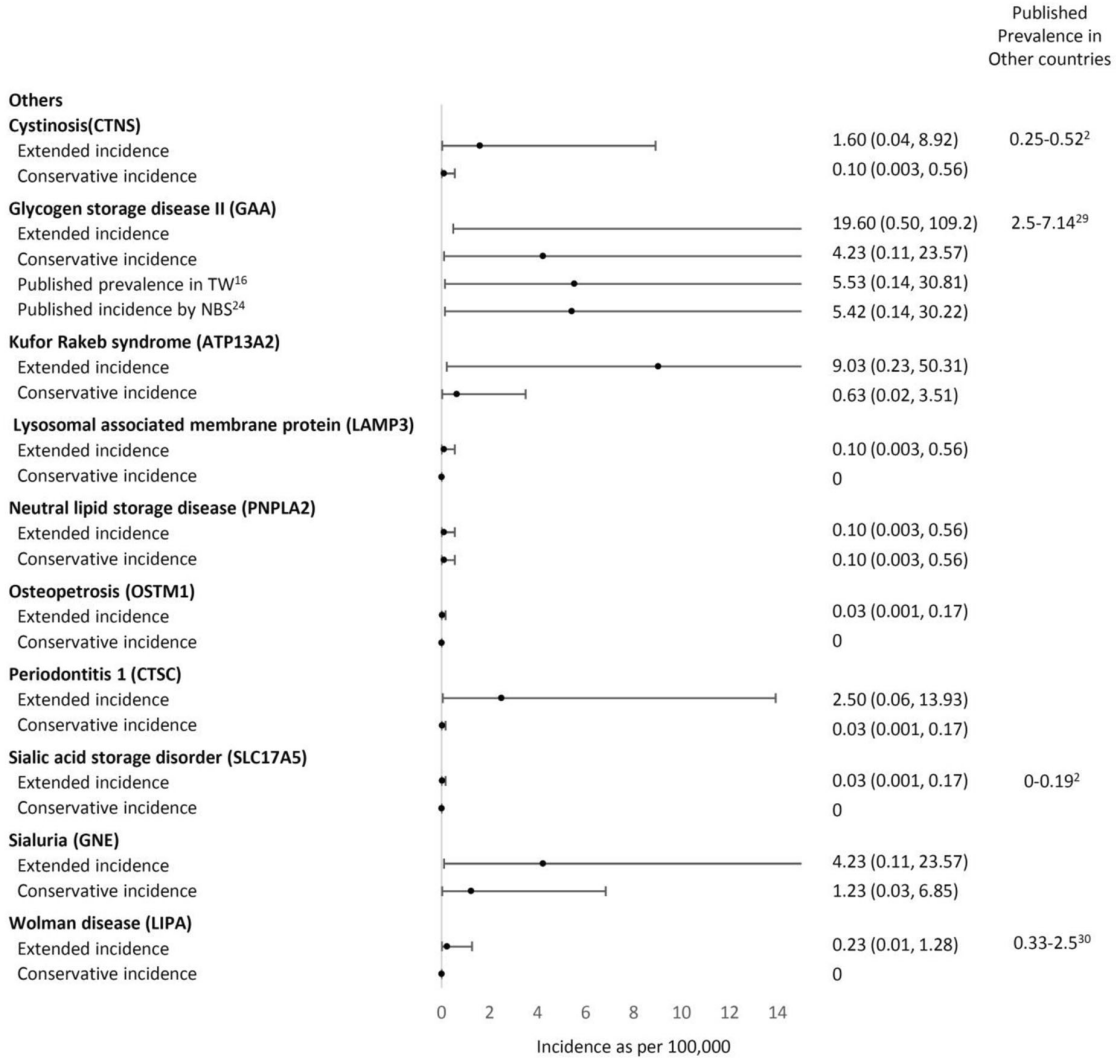
Comparison of the conservative and extended incidence with the known prevalence data. X-axis: incidence as per 100,000. *p value < 0.05.

A total of 51,963 variants in 74 genes related to LSDs were identified in data from 1495 Taiwanese individuals obtained from the TWB, with an average of 34 variants per gene (range 0–147). We included the 71 genes encoding for the autosomal recessive LSD and included variants with allele frequency <0.05 located in or near the exon region; 1003 variants remained for the subsequent analyses. A total of 270 variants in 53 genes were reported in ClinVar or the HGMD as “pathogenic” or unreported but with a high predicted severity score (severity score over 7 in the 13 prediction tools available from ANNOVAR). No variants were found in the following 18 genes: *NPC1, NPC2*, *ATG5, ATG7*, *BLOC1S3, CLCN5, OCRL, CLN4, CLN7, CTSK, DTNBP1, FIG4, GM2A, MCOLN1, SUMF1, mTORC1, SLC38A9*, and *SLC9A5*. We then curated the pathogenicity of these 270 variants according to the 2015 ACMG criteria (Supplementary Table 1)^12^. Twenty-seven variants were classified as pathogenic, 48 as likely pathogenic, 131 as VUS, and 64 as benign or likely benign (Figs. 1 and 2). Overall, 15 of the 53 genes demonstrated VUS but no known pathogenic or likely pathogenic variants. Another three demonstrated only benign or likely benign variants. A total of 21 unreported variants were identified in this cohort (Table 1). We calculated the estimated conservative disease incidence by including only pathogenic and likely pathogenic variants (P + LP) and the estimated extended incidence by incorporating VUS (P + LP + VUS). The calculated incidence for each LSD is listed in Fig. 3. The total incidence of autosomal recessive LSDs was 13 per 100,000 (95% CI 6.92–22.23) by conservative estimation and 94 per 100,000 (95% CI 75.96–115.03) by extended estimation.

**Table 1:** The unreported variants identified in Taiwanese individuals and the related disorders.

Gene	Chr	Start	End	Transcript	Exon	Nucleotide	Amino Acid	Disease	Taiwan Biobank	MAF	ACMG
*HYAL1*	3	50302735	50302735	NM_153281	exon4	c.221dupT	p.Y75Lfs*2	Mucopolysaccharidosis type IX	0.0005	0.0006	Likely pathogenic [PS1, PM3]
*HPS3*	3	149172202	149172202	NM_032383	exon17	c.C2995T	p.R999X	Hermansky-Pudlak syndrome 3	0.0005	0.0005	Pathogenic [PS1, PM3, PP4]
*MANBA*	4	102722993	102722993	NM_005908	exon4	c.427delC	p.R143Vfs*73	Beta-mannosidosis	0.0005	0.0005	Likely pathogenic [PS1, PM3]
*MFSD8*	4	127965131	127965131	NM_152778	exon2	c.G3C	p.M1?	Neuronal ceroid lipofuscinosis 7	0.0005	0.0005	Likely pathogenic [PS1, PM3]
*GUSB*	7	65970302	65970302	NM_000181	exon9	c.1455dupT	p.N486*	Mucopolysaccharidosis type VII	0.0005	0.0005	Likely pathogenic [PS1, PM3]
*ASAH1*	8	18063213	18063213	NM_177924	exon7	c.A475T	p.R159X	Farber lipogranulomatosis	0.0005	0.0005	Pathogenic [PS1, PM3, PP4]
*HGSNAT*	8	43140604	43140604	NM_152419	exon1	c.108_109insCCGCCACGAGGTGAGTGCACACCTCCTA	p.D40Gfs*30	Mucopolysaccharidosis type IIIC	0.0005	0.0005	Likely pathogenic [PS1, PM3]
*GNE*	9	36246215	36246215	NM_001128227	exon3	c.524dupA	p.D175Efs*30	Sialuria	0.0005	0.0005	Likely pathogenic [PS1, PM3]
*HPS6*	10	102067113	102067114	NM_024747	exon1	c.1639_1640del	p.L548Rfs*18	Hermansky-Pudlak syndrome 6	0.0005	0.0005	Likely pathogenic [PS1, PM3]
*PNPLA2*	11	821684	821684	NM_020376	exon3	c.G244A	p.G82S	Neutral lipid storage disease	0.0005	0.0005	Likely pathogenic [PS1, PM3]
*PNPLA2*	11	824737	824737	NM_020376	exon10	c.1390delC	p.A465Pfs*24	Neutral lipid storage disease	0.0005	0.0005	Likely pathogenic [PS1, PM3]
*SMPD1*	11	6391633	6391640	NM_000543	exon2	c.568_575del	p.P194Rfs*12	Niemann-Pick disease type A/B	0.0035	0.0035	Likely pathogenic [PS1, PM3]
*HPS5*	11	18291846	18291846	NM_181507	exon16	c.C2036A	p.S679X	Hermansky-Pudlak syndrome 5	0.0015	0.0015	Pathogenic [PS1, PM3, PP4]
*CTSF*	11	66566045	66566045	NM_003793	exon6	c.844delG	p.A282Lfs*64	Neuronal ceroid lipofuscinosis 13	0.0005	0.0005	Likely pathogenic [PS1, PM3]
*CTSC*	11	88296248	88296248	NM_001814	exon6	c.C774A	p.C258X	Periodontitis 1	0.0005	0.0005	Pathogenic [PS1, PM3, PP4]
*HEXA*	15	72350530	72350530	NM_000520	exon7	c.T793C	p.S265P	GM2-gangliosidosis/Tay-Sachs disease	0.0005	0.0005	Likely pathogenic [PS1, PM3]
*GNPTG*	16	1363060	1363060	NM_032520	exon11	c.887delG	p.G297Vfs*37	Mucolipidosis III gamma	0.0005	0.0005	Likely pathogenic [PS1, PM3]
*GALNS*	16	88832003	88832003	NM_000512	exon9	c.G997A	p.G333S	Mucopolysaccharidosis type IVA	0.0005	0.0005	Likely pathogenic [PS1, PM3]
*GALNS*	16	88856761	88856761	NM_000512	exon1	c.C117G	p.D39E	Mucopolysaccharidosis type IVA	0.0005	0.0005	Likely pathogenic [PS1, PM3]
*NAGLU*	17	42544009	42544009	NM_000263	exon6	c.2003delC	p.N669Tfs*138	Mucopolysaccharidosis type IIIB	0.0005	0.0005	Likely pathogenic [PS1, PM3]
*CTSA*	20	45898426	45898426	NM_000308	exon15	c.1420dupT	p.L475Pfs*17	Galactosialidosis	0.0005	0.0005	Likely pathogenic [PS1, PM3]

*ACMG* American College of Medical Genetics and Genomics, *MAF* maximum minor allele frequency from the Exome Aggregation Consortium (ExAC), 1000 Genomes, GenomAD, and Taiwan biobank database.

### The incidence of MPSs

We compared the current data with previously published prevalence data (Fig. 3) using retrospective clinical observation and newborn population screening data. Regarding MPSs, our conservative incidence estimation was 2.21 per 100,000 (95% CI: 0.056–12.313), which showed no statistical significance (*p* = 0.549) in comparison with the combined birth prevalence except for MPS II, which demonstrated an incidence of 0.97 per 100,000 live births^13^. The extended incidences of total MPS and MPS type IIIA, IIIC, and IVA were significantly higher than those shown in the incidence data reported by Lin et al. (Fig. 3)^13^. However, compared with the incidence observed in newborn screening for MPS type I, IIIB, and IVA^13^, our conservatively calculated incidence was significantly lower (Fig. 3).

### The incidence of Pompe disease

The incidence of Pompe was also assessed using newborn screening data^14^. The incidence (prevalence at birth) was reported to be 55 in 994,975 from 2005 to 2018 (5.53 per 100,000)^15^. Our conservative estimation for Pompe disease was 4.23, thus, did not significantly differ from the reported incidence (Fig. 3).

## Discussion

Here, using genetic data obtained from the TWB, we estimated that the combined incidence of 71 autosomal recessive LSDs is between 13 per 100,000 (pathologic and likely pathogenic variants) and 94 per 100,000 (pathologic and likely pathogenic variants and variants of unknown significance). This incidence range is considerably higher than the reported prevalence among clinical cases but similar to that obtained through newborn screening. LSDs are very rare, and diagnoses are often delayed or missed; therefore, an accurate estimation of the incidence of these diseases in Taiwan is challenging, if not impossible. Therefore, estimation methods from genome-wide sequencing databases, as conducted in this study, or unbiased population screening are alternative methods for understanding the true incidence of these diseases. Thus, these approaches assist in the development of policies that address the burden of rare diseases.

The conservative estimation data in the current study were more similar to the incidence rates observed in the clinic than the extended estimation data. For example, regarding MPS I, the conservative incidence estimate (0.03; 95% CI: 0.001–0.17) is similar to the published incidence in Taiwan (0.11; 95% CI: 0.003–0.61)^13^, confirming that this is an extremely rare disease. However, the extended and newborn screening incidences demonstrated a wider estimation range, implying that a milder or late-onset phenotype may exist that is not easily recognized by clinicians. Further understanding of the pathogenicity of VUS, either with functional or long-term follow-up data obtained through newborn screening, may further elucidate the true incidence of MPS I.

Although we analyzed limited genomic data in this study, the general incidence obtained is similar to that obtained in a previously published large-scale biobank study of the same population^16^. The carrier rate of Krabbe disease (*GALC* gene) in the previous study was estimated to be 1.67%, similar to the current study’s estimate (0.2-2.18%). Regarding mucolipidosis type II/III (*GNPTAB*), the previous estimate was 0.44%, and the current estimate is 0.3–1%, and the difference between the two estimates is not significant. The comparison of Pompe disease and GAA carrier incidence between the previous study and ours is more indirect, as the previous group calculated only the allele frequency (0.38%) of *GAA* causing infantile-onset Pompe disease among 103,106 individuals Taiwan^16^; however, we included late-onset and infantile-onset Pompe disease, yielding a conservative allele frequency of 0.65%. Overall, our data support validation using a small dataset instead of a large dataset such as the biobank. Since TWB 2.0 only contained 179 known disease-relevant regions^16^, the use of TWB 2.0 may decrease the ability to detect rare variants in rare diseases. However, the current study demonstrated no differences when using larger SNP chip datasets versus comprehensive whole-genome sequencing (WGS) data from a small population. It would be due to the fact that only exonic and nearby intronic variants were analyzed. Further validation will be required when more WGS data become available.

In this study, the allele frequency in Taiwanese individuals was too low to calculate the variant incidence for 18 among the 71 genes encoding for the autosomal recessive LSD, and an additional 18 genes among the rest of 53 genes without pathogenic variants were recorded. For example, in *NPC1* and *NPC2*, which cause Niemann-Pick disease type C, no *NPC1* variants were identified in the WGS data from the 1495 individuals in the TWB, and only one *NPC2* variant was identified. The *NPC2* variant was excluded because the severity score was 4 over 13. The published prevalence of Niemann-Pick disease type C is 0.25 per 100,000 in the United Arab Emirates and 2.2 per 100,000 in Portugal^2^., which converts to a carrier rate of at least 1 in 400. This range indicates that the variants should have been present among the 1,495 individuals studied here. Our current data demonstrate an even lower incidence of Niemann-Pick disease type C in Taiwan, although clinical cases have been reported^7^. The existence of selection bias, which is the prevalence of diseases only in specific populations, requires further study. Selection bias is less likely in our study because of Taiwan’s relatively homogenous Chinese-Han population^12^. We are not aware of any clustering of such LSD in specific populations in this country.

Many biobanks, such as the Global Biobank Meta-analysis Initiative (GBMI), UK Biobank, Estonian Biobank and China Biobank, have been established worldwide as a result of improvements in NGS techniques. Many researchers have tried used data from different biobanks to predict the risk or prevalence of different diseases. Most select likely pathogenic variants^16,17^ and use the Hardy–Weinberg equation to calculate the disease incidence, as in this study. Currently, most studies rely on biobank data to determine disease incidence and identify genetic and non-genetic factors contributing to various common chronic diseases. However, to date, no additional omics studies have been conducted. In the future, it would be highly valuable to organize further omics studies to delve deeper into the underlying mechanisms and molecular aspects of these diseases. Such studies could provide a more comprehensive understanding of the diseases’ complexities and potentially lead to more targeted and effective interventions. In addition, we estimated conservative and extended disease incidences due to the uncertainty of VUS curation and to better estimate the disease incidence range. Nevertheless, because biobanks are generated using different types of omics data, such as genotype arrays and WGS, additional caution should be taken when applying the resulting datasets to estimate disease prevalence. Furthermore, those biobanks, although population-based, may not represent the general population regarding sociodemographic or health-related characteristics^18^ and may not be a suitable resource for determining disease prevalence and incidence rates. UK BioBank has released 50,000 exomes^19^ and will add an additional 200,000 exomes to become the largest open-access resource of WES data linked to health records. A better understanding of rare disease incidence is expected in the future following analyses of a larger WES dataset.

Our study did not assess X-linked LSDs because the equation for X-linked disorders requires different interpretation methods, especially for those diseases with late-onset phenotypes. For example, the newborn screening for Fabry disease by enzyme assay revealed an incidence rate of 1 in 1250 among Taiwanese males^20^, most of whom had the *GLA* IVS4 + 919 G > A variant. The incidence rate of the *GLA* IVS4 + 919 G > A variant is estimated to be 1 in 600 among newborns^21^; however, it is unknown if those individuals participated without bias in the small WGS dataset used in this study; therefore, we did not include X-linked LSD.

Another limitation of our study is associated with the short-read WGS method. For example, the *GBA* gene recombines with its pseudogene; thus, it is challenging to determine where the variants are located accurately using WGS. Therefore, we could only roughly estimate the incidence of Gaucher disease. However, in the newborn screening data, the incidence range was similar to that estimated from the dataset^22^. Thus, we consider that our results provide useful information for estimating the burden of autosomal recessive LSD, although further clarification, such as improving the methods or data from biochemical screening, may be warranted for specific conditions.

Finally, although we used the WGS database, mutations in deep introns not regarded as critical for splicing may have been missed, and copy number changes were not reported. However, we could not demonstrate a significant difference in incidence when comparing our data to available NGS data regarding biochemical and protein levels, implying the minimal impact of using such genomic data for estimation. When calculating incidence, the possibility of in-*cis v*ariants was not considered; thus, the incidence may have been overestimated. The increasing acceptance of preconception carrier screening could also influence the clinical incidence since prenatal diagnosis and abortion if the fetus is found to be affected are permitted in Taiwanese culture. Such incidence drift has been observed in thalassemia and spinal muscular atrophy carrier screening, which are performed widely in Taiwan^23^.

In conclusion, the current study generated useful incidence data regarding LSDs in Taiwan. Our curated, conservative estimation of incidence could guide public health measures in calculating disease or drug burdens. Our extended estimation could also facilitate newborn and high-risk screening. Incidence estimation from genomic data will improve further as the clinical significance of variants becomes better understood.

## Methods

### The Taiwan Biobank

The TWB is a government-supported database that facilitates biomedical genetic research in the Taiwanese population (https://www.twbiobank.org.tw). The TWB was initiated as an ongoing prospective study in 2012 with a target sample size of 200,000 individuals aged 20–70 with no prior cancer diagnosis. At recruitment, participants provided a written informed consent and had their baseline data collected. As of 30 November 2022, 184,577 volunteers have participated in biobanking, and 45,439 have completed the first follow-up round.

### Whole-genome sequencing

In the current study, we used WGS data obtained from 1495 Taiwanese individuals in the TWB. The WGS data were generated using Illumina platforms, and the experiments and analyses were conducted by Genomics BioSci & Tech Co., Ltd. DNA was extracted from blood samples. Sequenced was done by the Illumina Hi-Seq 2500 (2 × 150 bp paired-end) with output of 90GB and an average coverage depth of 30x. Raw reads were mapped to hg38 genome reference by BWA-MEM2 and variants were called by the Genome Analysis Toolkit (GATK) haplotypecaller. Subsequently, we employed the WGS data to calculate the incidence of LSDs.

### LSD gene selection

We selected the genes from the lysosomal disorder and mucopolysaccharidosis panel in Blueprint genetics (https://blueprintgenetics.com/). Mutations in a total of 74 genes are known to cause LSDs. X chromosome variants were not included, including those in the *IDS*, *LAMP2*, and *GLA* genes.

### Curation of variants and estimation of incidence

We first included single nucleotide variants (SNVs) in the exon and exon/intron border and small indel variants; the allele frequency of all variants in the TWB was ≤0.05. We then included variants according to the following criteria: (1) Reported in ClinVar as pathogenic or in the Human Gene Mutation Database (HGMD) as disease-causing mutations (DM) or possible/probable disease-causing mutations (DM?); or (2) unreported in ClinVar or the HGMD with a severity score exceeding 7 in the 13 prediction tools [Sorting Intolerant From Tolerant (SIFT), PolyPhen-2 (Polymorphism Phenotyping v2) HDIV, PolyPhen-2 HVAR, LRT (Likelihood Ratio Test), Mutation Taster, Mutation Assessor, FATHMM (Functional Analysis through Hidden Markov Models), FATHMM-MKL, Provean (Protein Variation Effect Analyzer), CADD (Combined Annotation–Dependent Depletion), MetaSVM, MetaLR, Mendelian Clinically Applicable Pathogenicity (M-CAP)].

The pathogenicity of the variants was determined according to American College of Medical Genetics and Genomics (ACMG) guidelines^12^. Risk alleles were defined as pathogenic (P), likely pathogenic (LP), or variants of unknown significance (VUS). Gene-specific risk allele frequency (q) was defined as the sum of the frequency of all variants in the indicated gene. Linkage between variants within a gene was not assessed. Therefore, the probability of having a risk allele for a disease in the haploid genome of a population was q and that of not having a risk allele was Q = 1 − q. The carrier rate was then calculated as 2 × Q × q based on Hardy–Weinberg equilibrium. The disease incidence was calculated as q^2^. The calculated LSD disease incidences were then compared with real-world epidemiological data.

### Statistics

The statistical analyses were performed using MedCalc® Statistical Software version 20.2 (MedCalc Software Ltd, Ostend, Belgium; https://www.medcalc.org; 2022). Comparisons of two rates were used to calculate the 95% confidence interval (95% CI) and *p* value between the estimated and the reported incidence. A *p* value < 0.05 was considered to indicate significance.

### Reporting summary

Further information on research design is available in the Nature Research Reporting Summary linked to this article.

### Supplementary information

Supplementary Table 1

Reporting Summary

## Data Availability

All structured data generated or analyzed during this study are included in this published article and its supplementary information files. The dataset generated during and/or analyzed during the current study are available from the corresponding author on reasonable request.

## Code availability

All computational tools used in this study are available for download as commercial or open-source software and are detailed in Methods.

## References

[1] Meikle PJ, Hopwood JJ, Clague AE, Carey WF (1999). Prevalence of lysosomal storage disorders. JAMA.

[2] Kingma SD, Bodamer OA, Wijburg FA (2015). Epidemiology and diagnosis of lysosomal storage disorders; challenges of screening. Best. Pract. Res Clin. Endocrinol. Metab..

[3] Pinto R (2004). Prevalence of lysosomal storage diseases in Portugal. Eur. J. Hum. Genet.

[4] Puckett Y, Mallorga-Hernandez A, Montano AM (2021). Epidemiology of mucopolysaccharidoses (MPS) in United States: challenges and opportunities. Orphanet. J. Rare Dis..

[5] Chen X (2016). Demographic characteristics and distribution of lysosomal storage disorder subtypes in Eastern China. J. Hum. Genet..

[6] Almannai M, Marom R, Sutton VR (2016). Newborn screening: a review of history, recent advancements, and future perspectives in the era of next generation sequencing. Curr. Opin. Pediatr..

[7] Chien YH, Hwu WL, Lee NC (2013). Pompe disease: early diagnosis and early treatment make a difference. Pediatr. Neonatol..

[8] Chiang SC (2018). Performance of the four-plex tandem mass spectrometry lysosomal storage disease newborn screening test: the necessity of adding a 2nd tier test for Pompe disease. Int. J. Neonatal Screen.

[9] Dabbous O (2023). Valuation of treatments for rare diseases: a systematic literature review of societal preference studies. Adv. Ther..

[10] Barton AR, Hujoel MLA, Mukamel RE, Sherman MA, Loh PR (2022). A spectrum of recessiveness among Mendelian disease variants in UK Biobank. Am. J. Hum. Genet..

[11] Schrodi SJ (2015). Prevalence estimation for monogenic autosomal recessive diseases using population-based genetic data. Hum. Genet..

[12] Richards S (2015). Standards and guidelines for the interpretation of sequence variants: a joint consensus recommendation of the American College of Medical Genetics and Genomics and the Association for Molecular Pathology. Genet. Med..

[13] Lin HY (2009). Incidence of the mucopolysaccharidoses in Taiwan, 1984-2004. Am. J. Med. Genet. A.

[14] Chien YH (2008). Early detection of Pompe disease by newborn screening is feasible: results from the Taiwan screening program. Pediatrics.

[15] Lee NC (2022). Outcome of later-onset Pompe disease identified through newborn screening. J. Pediatr..

[16] Wei CY (2021). Genetic profiles of 103,106 individuals in the Taiwan Biobank provide insights into the health and history of Han Chinese. NPJ Genom. Med..

[17] Gilchrist M (2023). Prevalence of Fabry disease-causing variants in the UK Biobank. J. Med. Genet..

[18] Fry A (2017). Comparison of sociodemographic and health-related characteristics of UK Biobank participants with those of the general population. Am. J. Epidemiol..

[19] Van Hout CV (2020). Exome sequencing and characterization of 49,960 individuals in the UK Biobank. Nature.

[20] Hwu WL (2009). Newborn screening for Fabry disease in Taiwan reveals a high incidence of the later-onset GLA mutation c.936+919G>A (IVS4+919G>A). Hum. Mutat..

[21] Chien YH, Lee NC, Chiang SC, Desnick RJ, Hwu WL (2012). Fabry disease: incidence of the common later-onset alpha-galactosidase A IVS4+919G->A mutation in Taiwanese newborns–superiority of DNA-based to enzyme-based newborn screening for common mutations. Mol. Med..

[22] Liao HC (2014). Detecting multiple lysosomal storage diseases by tandem mass spectrometry–a national newborn screening program in Taiwan. Clin. Chim. Acta.

[23] Chern JP (2008). Beta-thalassemia major births after national screening program in Taiwan. Pediatr. Blood Cancer.

[24] Chien YH (2020). Newborn screening for Morquio disease and other lysosomal storage diseases: results from the 8-plex assay for 70,000 newborns. Orphanet J. Rare Dis..

[25] Chuang CK (2021). Nationwide newborn screening program for Mucopolysaccharidoses in Taiwan and an update of the “gold standard” criteria required to make a confirmatory diagnosis. Diagnostics.

[26] Chan MJ (2019). Taiwan national newborn screening program by tandem mass spectrometry for Mucopolysaccharidoses types I, II, and VI. J. Pediatr..

[27] De Jesus Rojas W, Young LR (2020). Hermansky-Pudlak Syndrome. Semin. Respir. Crit. Care Med..

[28] Santorelli FM (2013). Molecular epidemiology of childhood neuronal ceroid-lipofuscinosis in Italy. Orphanet. J. Rare Dis..

[29] Dasouki M (2014). Pompe disease: literature review and case series. Neurol. Clin..

[30] Pericleous M, Kelly C, Wang T, Livingstone C, Ala A (2017). Wolman’s disease and cholesteryl ester storage disorder: the phenotypic spectrum of lysosomal acid lipase deficiency. Lancet Gastroenterol. Hepatol..

