# Supplementary material for: Curated incidence of lysosomal storage diseases from the Taiwan Biobank
Source: NPJ Genom Med. 2023 Sep 23;8:27. doi: 10.1038/s41525-023-00372-x (PMC10517920; doi:10.1038/s41525-023-00372-x)
Supplement: Supplementary file 1 — Supplementary Table 1 [file 41525_2023_372_MOESM1_ESM.docx]

Supplementary Table 1. Total 270 variants

| **Gene** | **Chr** | **Start** | **End** | **Transcript** | **Exon** | **Nucleotide** | **Amino Acid** | **dbSNP ID** | **Taiwan Biobank** | **AMF** | **ACMG** |
| --- | --- | --- | --- | --- | --- | --- | --- | --- | --- | --- | --- |
| *ATP13A2* | 1 | 16986825 | 16986825 | NM_022089 | exon27 | c.G3215A | p.R1072H | rs371220869 | 0.0005 | 0.0005 | Likely benign |
| *ATP13A2* | 1 | 16987089 | 16987089 | NM_022089 | exon26 | c.G3040A | p.G1014S | rs202166353 | 0.0005 | 0.0012 | Uncertain significance |
| *ATP13A2* | 1 | 16988461 | 16988461 | NM_022089 | exon24 | c.A2623T | p.M875L | rs767865035 | 0.0005 | 0.0005 | Uncertain significance |
| *ATP13A2* | 1 | 16989746 | 16989746 | NM_022089 | exon23 | c.G2554T | p.A852S | rs747242869 | 0.0005 | 0.0005 | Uncertain significance |
| *ATP13A2* | 1 | 16991749 | 16991749 | NM_022089 | exon20 | c.G2236A | p.A746T | rs147277743 | 0.001 | 0.0017 | Uncertain significance |
| *ATP13A2* | 1 | 16991787 | 16991787 | NM_022089 | exon20 | c.C2198T | p.T733M | rs201883464 | 0.0025 | 0.0042 | Uncertain significance |
| *ATP13A2* | 1 | 16997136 | 16997136 | NM_022089 | exon12 | c.G1079A | p.G360E | rs566918264 | 0.0015 | 0.0031 | Uncertain significance |
| *ATP13A2* | 1 | 17000407 | 17000407 | NM_022089 | exon9 | c.C833A | p.T278N | rs773768330 | 0.0005 | 0.0014 | Likely benign |
| *ATP13A2* | 1 | 17000419 | 17000419 | NM_022089 | exon9 | c.C821T | p.S274L | . | 0.0005 | 0.0005 | Uncertain significance |
| *ATP13A2* | 1 | 17000495 | 17000495 | NM_022089 | exon9 | c.G745A | p.A249T | rs199661793 | 0.0025 | 0.0025 | Likely pathogenic |
| *FUCA1* | 1 | 23846097 | 23846097 | NM_000147 | exon7 | c.C1237T | p.P413S | rs776717041 | 0.0005 | 0.0011 | Uncertain significance |
| *FUCA1* | 1 | 23859895 | 23859895 | NM_000147 | exon4 | c.671delC | p.P224Lfs*3 | . | 0.0005 | 0.0005 | Likely pathogenic |
| *FUCA1* | 1 | 23863264 | 23863264 | NM_000147 | exon3 | c.C532T | p.R178C | rs149540896 | 0.0005 | 0.0033 | Uncertain significance |
| *FUCA1* | 1 | 23865526 | 23865526 | NM_000147 | exon2 | c.T489A | p.D163E | . | 0.0005 | 0.0005 | Uncertain significance |
| *FUCA1* | 1 | 23865530 | 23865530 | NM_000147 | exon2 | c.G485A | p.R162Q | rs372045495 | 0.0005 | 0.0006 | Uncertain significance |
| *FUCA1* | 1 | 23867927 | 23867927 | NM_000147 | exon1 | c.G360C | p.W120C | rs758745364 | 0.001 | 0.0036 | Uncertain significance |
| *PPT1* | 1 | 40076858 | 40076858 | NM_000310 | exon8 | c.C782A | p.T261N | . | 0.0005 | 0.0005 | Uncertain significance |
| *PPT1* | 1 | 40092479 | 40092479 | NM_000310 | exon2 | c.G153C | p.M51I | . | 0.0005 | 0.0005 | Uncertain significance |
| *GBA* | 1 | 155235727 | 155235727 | NM_001005741 | exon10 | c.G1342C | p.D448H | rs1064651 | 0.0005 | 0.0006 | Likely pathogenic |
| *GBA* | 1 | 155235790 | 155235790 | NM_001005741 | exon10 | c.G1279A | p.E427K | rs149171124 | 0.0005 | 0.0005 | Uncertain significance |
| *GBA* | 1 | 155238290 | 155238290 | NM_001005741 | exon7 | c.G605A | p.R202Q | rs398123531 | 0.004 | 0.004 | Uncertain significance |
| *GBA* | 1 | 155240687 | 155240687 | NM_001005741 | exon3 | c.A58G | p.I20V | rs143187997 | 0.0005 | 0.0038 | Uncertain significance |
| *GLB1* | 3 | 32997077 | 32997077 | NM_000404 | exon16 | c.A2002T | p.K668X | rs758168173 | 0.0005 | 0.0012 | Likely pathogenic |
| *GLB1* | 3 | 33018485 | 33018485 | NM_000404 | exon13 | c.A1310G | p.N437S | rs202237232 | 0.0005 | 0.0085 | Likely benign |
| *GLB1* | 3 | 33021571 | 33021571 | NM_000404 | exon12 | c.A1228C | p.K410Q | rs779369366 | 0.001 | 0.001 | Likely benign |
| *GLB1* | 3 | 33024313 | 33024313 | NM_000404 | exon11 | c.C1081T | p.P361S | . | 0.0005 | 0.0005 | Uncertain significance |
| *GLB1* | 3 | 33051895 | 33051895 | NM_000404 | exon8 | c.C902T | p.A301V | rs750531880 | 0.0005 | 0.0005 | Likely pathogenic |
| *GLB1* | 3 | 33058106 | 33058106 | NM_000404 | exon6 | c.C716T | p.T239M | rs746766232 | 0.0005 | 0.0005 | Likely pathogenic |
| *GLB1* | 3 | 33068236 | 33068236 | NM_000404 | exon4 | c.G451A | p.D151N | rs375582374 | 0.0005 | 0.0005 | Likely pathogenic |
| *GLB1* | 3 | 33068241 | 33068241 | NM_000404 | exon4 | c.C446T | p.S149F | rs778700089 | 0.0005 | 0.0005 | Likely pathogenic |
| *HYAL1* | 3 | 50302085 | 50302085 | NM_153281 | exon4 | c.A872G | p.Y291C | rs782313024 | 0.0005 | 0.0005 | Uncertain significance |
| *HYAL1* | 3 | 50302509 | 50302509 | NM_153281 | exon4 | c.C448T | p.R150C | rs781875133 | 0.0005 | 0.0005 | Uncertain significance |
| *HYAL1* | 3 | 50302735 | 50302735 | NM_153281 | exon4 | c.221dupT | p.Y75Lfs*2 | . | 0.0005 | 0.0006 | Likely pathogenic |
| *HPS3* | 3 | 149157357 | 149157357 | NM_032383 | exon9 | c.A1517G | p.Y506C | rs375394383 | 0.0005 | 0.0006 | Uncertain significance |
| *HPS3* | 3 | 149158812 | 149158812 | NM_032383 | exon10 | c.C1838G | p.S613X | rs755083879 | 0.0005 | 0.0012 | Pathogenic |
| *HPS3* | 3 | 149162248 | 149162249 | NM_032383 | exon12 | c.2207_2208del | p.Q737Afs*20 | rs745457191 | 0.0005 | 0.0005 | Pathogenic |
| *HPS3* | 3 | 149167142 | 149167142 | NM_032383 | exon15 | c.C2698T | p.R900C | rs751160941 | 0.0005 | 0.0005 | Likely benign |
| *HPS3* | 3 | 149172202 | 149172202 | NM_032383 | exon17 | c.C2995T | p.R999X | . | 0.0005 | 0.0005 | Pathogenic |
| *LAMP3* | 3 | 183153714 | 183153714 | NM_014398 | exon2 | c.G727A | p.G243R | . | 0.0005 | 0.0005 | Uncertain significance |
| *LAMP3* | 3 | 183153752 | 183153752 | NM_014398 | exon2 | c.T689C | p.V230A | rs749779464 | 0.0005 | 0.0005 | Uncertain significance |
| *IDUA* | 4 | 987086 | 987086 | NM_000203 | exon1 | c.T2C | p.M1? | rs753767675 | 0.0005 | 0.0005 | Pathogenic |
| *IDUA* | 4 | 1001545 | 1001545 | NM_000203 | exon5 | c.G571C | p.V191L | rs746908649 | 0.0005 | 0.0018 | Likely benign |
| *IDUA* | 4 | 1002426 | 1002426 | NM_000203 | exon8 | c.C1130T | p.P377L | rs546808806 | 0.0005 | 0.0006 | Uncertain significance |
| *IDUA* | 4 | 1002465 | 1002465 | NM_000203 | exon8 | c.T1169C | p.M390T | . | 0.0005 | 0.0005 | Uncertain significance |
| *IDUA* | 4 | 1002767 | 1002767 | NM_000203 | exon9 | c.G1225C | p.G409R | rs11934801 | 0.015 | 0.0766 | Benign |
| *IDUA* | 4 | 1003345 | 1003345 | NM_000203 | exon11 | c.G1525A | p.D509N | . | 0.0005 | 0.0005 | Likely benign |
| *IDUA* | 4 | 1003417 | 1003417 | NM_000203 | exon11 | c.C1597T | p.P533S | rs374779600 | 0.0005 | 0.0005 | Uncertain significance |
| *MANBA* | 4 | 102632224 | 102632224 | NM_005908 | exon17 | c.G2473T | p.A825S | rs182869272 | 0.005 | 0.0068 | Benign |
| *MANBA* | 4 | 102634957 | 102634957 | NM_005908 | exon16 | c.T2246A | p.L749H | rs142248415 | 0.032 | 0.032 | Likely benign |
| *MANBA* | 4 | 102639814 | 102639814 | NM_005908 | exon14 | c.G1913A | p.R638H | rs781584789 | 0.002 | 0.0031 | Uncertain significance |
| *MANBA* | 4 | 102650697 | 102650697 | NM_005908 | exon13 | c.C1709T | p.S570L | rs542071291 | 0.002 | 0.002 | Likely benign |
| *MANBA* | 4 | 102664831 | 102664831 | NM_005908 | exon11 | c.C1339T | p.P447S | rs755785845 | 0.001 | 0.001 | Likely benign |
| *MANBA* | 4 | 102673921 | 102673921 | NM_005908 | exon8 | c.G1110A | p.E370E |  | 0.0005 | 0.0005 | Uncertain significance |
| *MANBA* | 4 | 102673965 | 102673965 | NM_005908 | exon8 | c.A1066G | p.N356D | rs769666791 | 0.0005 | 0.0005 | Uncertain significance |
| *MANBA* | 4 | 102722993 | 102722993 | NM_005908 | exon4 | c.427delC | p.R143Vfs*73 | . | 0.0005 | 0.0005 | Likely pathogenic |
| *MANBA* | 4 | 102722890 | 102722890 | NM_005908 | exon4 | c.A530G | p.H177R | rs750914797 | 0.0035 | 0.0049 | Likely benign |
| *MANBA* | 4 | 102723034 | 102723034 | NM_005908 | exon4 | c.A386G | p.D129G | rs777157327 | 0.0005 | 0.0012 | Likely benign |
| *MANBA* | 4 | 102723893 | 102723893 | NM_005908 | exon3 | c.T347C | p.I116T | rs775841860 | 0.0005 | 0.0005 | Likely benign |
| *MFSD8* | 4 | 127920743 | 127920743 | NM_152778 | exon13 | c.C1444T | p.R482X | rs724159971 | 0.0005 | 0.0006 | Pathogenic |
| *MFSD8* | 4 | 127921959 | 127921959 | NM_152778 | exon11 | c.G1003A | p.G335S | . | 0.0005 | 0.0005 | Uncertain significance |
| *MFSD8* | 4 | 127943935 | 127943935 | NM_152778 | exon5 | c.G256A | p.G86S | rs552923962 | 0.001 | 0.001 | Likely benign |
| *MFSD8* | 4 | 127943973 | 127943973 | NM_152778 | exon5 | c.217dupA | p.T73Nfs*12 | rs796052752 | 0.0005 | 0.0005 | Pathogenic |
| *MFSD8* | 4 | 127965131 | 127965131 | NM_152778 | exon2 | c.G3C | p.M1? | . | 0.0005 | 0.0005 | Likely pathogenic |
| *AGA* | 4 | 177439605 | 177439605 | NM_000027 | exon3 | c.C365G | p.T122R | rs771563230 | 0.0005 | 0.0005 | Uncertain significance |
| *HEXB* | 5 | 74713567 | 74713567 | NM_000521 | exon7 | c.C833T | p.A278V | . | 0.0005 | 0.0005 | Uncertain significance |
| *HEXB* | 5 | 74721149 | 74721149 | NM_000521 | exon14 | c.G1645A | p.G549R | rs398123448 | 0.0005 | 0.0005 | Uncertain significance |
| *AP3B1* | 5 | 78002934 | 78002934 | NM_003664 | exon27 | c.C3253T | p.R1085W | . | 0.0005 | 0.0005 | Likely benign |
| *AP3B1* | 5 | 78002990 | 78002990 | NM_003664 | exon27 | c.C3197T | p.S1066F | rs764100439 | 0.001 | 0.0037 | Uncertain significance |
| *AP3B1* | 5 | 78175804 | 78175804 | NM_003664 | exon10 | c.A1075G | p.T359A | rs148160411 | 0.0015 | 0.0015 | Uncertain significance |
| *ARSB* | 5 | 78839358 | 78839358 | NM_000046 | exon6 | c.C1211T | p.P404L | rs200665696 | 0.0005 | 0.0005 | Likely benign |
| *ARSB* | 5 | 78839372 | 78839372 | NM_000046 | exon6 | c.C1197G | p.F399L | rs200793396 | 0.0005 | 0.0005 | Likely pathogenic |
| *ARSB* | 5 | 78969188 | 78969188 | NM_000046 | exon2 | c.G317A | p.R106H | rs150087888 | 0.001 | 0.0086 | Likely benign |
| *ARSB* | 5 | 78985058 | 78985058 | NM_000046 | exon1 | c.G191A | p.G64D | rs755330829 | 0.0005 | 0.0005 | Uncertain significance |
| *ARSB* | 5 | 78985068 | 78985068 | NM_000046 | exon1 | c.G181A | p.G61S | rs375647920 | 0.0005 | 0.0012 | Uncertain significance |
| *NEU1* | 6 | 31859849 | 31859849 | NM_000434 | exon6 | c.T1118C | p.L373P | . | 0.0005 | 0.0005 | Uncertain significance |
| *NEU1* | 6 | 31860159 | 31860159 | NM_000434 | exon5 | c.C904G | p.L302V | rs557409640 | 0.0005 | 0.0023 | Uncertain significance |
| *NEU1* | 6 | 31861259 | 31861259 | NM_000434 | exon3 | c.A544G | p.S182G | rs398123392 | 0.002 | 0.002 | Uncertain significance |
| *SLC17A5* | 6 | 73600377 | 73600377 | NM_012434 | exon10 | c.G1324A | p.V442I | rs74360232 | 0.0095 | 0.0095 | Likely benign |
| *SLC17A5* | 6 | 73641843 | 73641843 | NM_012434 | exon3 | c.C373T | p.P125S | rs754164697 | 0.0005 | 0.0006 | Likely benign |
| *SLC17A5* | 6 | 73641852 | 73641852 | NM_012434 | exon3 | c.A364G | p.T122A | . | 0.0005 | 0.0005 | Uncertain significance |
| *OSTM1* | 6 | 108051123 | 108051123 | NM_014028 | exon4 | c.C691A | p.L231M | . | 0.0005 | 0.0005 | Uncertain significance |
| *GUSB* | 7 | 65961051 | 65961051 | NM_000181 | exon12 | c.T1802G | p.V601G | rs762241590 | 0.008 | 0.008 | Uncertain significance |
| *GUSB* | 7 | 65961063 | 65961063 | NM_000181 | exon12 | c.C1790T | p.S597L | . | 0.0005 | 0.0005 | Uncertain significance |
| *GUSB* | 7 | 65970302 | 65970302 | NM_000181 | exon9 | c.1455dupT | p.N486* | . | 0.0005 | 0.0005 | Likely pathogenic |
| *GUSB* | 7 | 65974557 | 65974557 | NM_000181 | exon7 | c.G1213A | p.D405N | rs200149500 | 0.0015 | 0.0016 | Likely benign |
| *GUSB* | 7 | 65974686 | 65974686 | NM_000181 | exon7 | c.G1084A | p.D362N | rs398123234 | 0.0005 | 0.0005 | Likely pathogenic |
| *GUSB* | 7 | 65979808 | 65979808 | NM_000181 | exon3 | c.G500A | p.R167Q | . | 0.0005 | 0.0005 | Uncertain significance |
| *CLN8* | 8 | 1771428 | 1771428 | NM_018941 | exon2 | c.A374G | p.N125S | rs142269885 | 0.0005 | 0.0022 | Likely benign |
| *ASAH1* | 8 | 18057617 | 18057617 | NM_177924 | exon14 | c.G1105A | p.V369I | rs17636067 | 0.0345 | 0.0422 | Likely benign |
| *ASAH1* | 8 | 18061712 | 18061712 | NM_177924 | exon9 | c.G677C | p.R226P | . | 0.0005 | 0.0006 | Pathogenic |
| *ASAH1* | 8 | 18063213 | 18063213 | NM_177924 | exon7 | c.A475T | p.R159X | . | 0.0005 | 0.0005 | Pathogenic |
| *ASAH1* | 8 | 18063219 | 18063219 | NM_177924 | exon7 | c.C469T | p.H157Y | rs757410765 | 0.0005 | 0.0006 | Likely benign |
| *ASAH1* | 8 | 18067270 | 18067270 | NM_177924 | exon5 | c.C332G | p.P111R | . | 0.0005 | 0.0005 | Uncertain significance |
| *ASAH1* | 8 | 18071346 | 18071346 | NM_177924 | exon3 | c.C170T | p.P57L | rs11538152 | 0.002 | 0.0031 | Likely benign |
| *HGSNAT* | 8 | 43140604 | 43140604 | NM_152419 | exon1 | c.108_109insCCGCCACGAGGTGAGTGCACACCTCCTA | p.D40Gfs*30 | . | 0.0005 | 0.0005 | Likely pathogenic |
| *HGSNAT* | 8 | 43146975 | 43146975 | NM_152419 | exon2 | c.T146C | p.L49P | . | 0.0005 | 0.0005 | Uncertain significance |
| *HGSNAT* | 8 | 43159026 | 43159026 | NM_152419 | exon4 | c.C475T | p.P159S | rs573840743 | 0.0015 | 0.0043 | Likely benign |
| *HGSNAT* | 8 | 43178130 | 43178130 | NM_152419 | exon10 | c.G908A | p.R303Q | rs368646796 | 0.0005 | 0.0009 | Likely benign |
| *HGSNAT* | 8 | 43182162 | 43182162 | NM_152419 | exon11 | c.C1030T | p.R344C | rs121908285 | 0.001 | 0.001 | Likely pathogenic |
| *GNE* | 9 | 36217478 | 36217478 | NM_001128227 | exon12 | c.C2149G | p.Q717E | . | 0.0005 | 0.0005 | Uncertain significance |
| *GNE* | 9 | 36218261 | 36218261 | NM_001128227 | exon11 | c.G1948C | p.D650H | . | 0.0005 | 0.0005 | Uncertain significance |
| *GNE* | 9 | 36219969 | 36219969 | NM_001128227 | exon10 | c.T1778C | p.F593S | . | 0.0005 | 0.0005 | Uncertain significance |
| *GNE* | 9 | 36222839 | 36222839 | NM_001128227 | exon9 | c.C1664T | p.A555V | rs764698870 | 0.001 | 0.001 | Pathogenic |
| *GNE* | 9 | 36222885 | 36222885 | NM_001128227 | exon9 | c.C1618T | p.H540Y | rs754892377 | 0.001 | 0.001 | Uncertain significance |
| *GNE* | 9 | 36233986 | 36233986 | NM_001128227 | exon5 | c.C1009T | p.R337X | . | 0.0005 | 0.0005 | Pathogenic |
| *GNE* | 9 | 36236879 | 36236879 | NM_001128227 | exon4 | c.T815G | p.I272S | rs759945787 | 0.0005 | 0.0005 | Uncertain significance |
| *GNE* | 9 | 36246120 | 36246120 | NM_001128227 | exon3 | c.A620T | p.D207V | rs139425890 | 0.001 | 0.001 | Pathogenic |
| *GNE* | 9 | 36246215 | 36246215 | NM_001128227 | exon3 | c.524dupA | p.D175Efs*30 | . | 0.0005 | 0.0005 | Likely pathogenic |
| *GNE* | 9 | 36249325 | 36249325 | NM_001128227 | exon2 | c.C124T | p.R42W | rs769716748 | 0.0005 | 0.0005 | Likely pathogenic |
| *PSAP* | 10 | 71819599 | 71819599 | NM_002778 | exon11 | c.G1216A | p.G406S | rs747485660 | 0.0005 | 0.0005 | Uncertain significance |
| *PSAP* | 10 | 71828087 | 71828087 | NM_002778 | exon6 | c.C647T | p.S216F | . | 0.0005 | 0.0005 | Uncertain significance |
| *PSAP* | 10 | 71828145 | 71828145 | NM_002778 | exon6 | c.G589T | p.V197F | rs191952316 | 0.0005 | 0.0005 | Uncertain significance |
| *PSAP* | 10 | 71828921 | 71828921 | NM_002778 | exon5 | c.C532G | p.L178V | . | 0.0005 | 0.0005 | Uncertain significance |
| *LIPA* | 10 | 89214844 | 89214844 | NM_000235 | exon10 | c.T1184G | p.M395R | . | 0.0005 | 0.0005 | Uncertain significance |
| *LIPA* | 10 | 89214895 | 89214895 | NM_000235 | exon10 | c.T1133C | p.I378T | rs778013279 | 0.0005 | 0.0005 | Uncertain significance |
| *LIPA* | 10 | 89225176 | 89225176 | NM_000235 | exon6 | c.T591G | p.F197L | . | 0.0005 | 0.0005 | Uncertain significance |
| *HPS1* | 10 | 98420136 | 98420136 | NM_000195 | exon18 | c.C1766T | p.A589V | rs528827909 | 0.0025 | 0.0025 | Uncertain significance |
| *HPS1* | 10 | 98431282 | 98431282 | NM_000195 | exon7 | c.C517T | p.R173X | rs538274657 | 0.0005 | 0.0005 | Pathogenic |
| *HPS1* | 10 | 98435648 | 98435648 | NM_000195 | exon4 | c.A242T | p.Y81F | rs188320187 | 0.001 | 0.0018 | Likely benign |
| *HPS1* | 10 | 98443232 | 98443232 | NM_000195 | exon3 | c.C9A | p.C3X | . | 0.0005 | 0.0005 | Pathogenic |
| *HPS6* | 10 | 102065523 | 102065523 | NM_024747 | exon1 | c.49_50insGCGGC | p.L22Rfs*33 | rs752399257 | 0.0005 | 0.0005 | Likely pathogenic |
| *HPS6* | 10 | 102066297 | 102066297 | NM_024747 | exon1 | c.C823T | p.P275S | rs756325364 | 0.0005 | 0.0006 | Uncertain significance |
| *HPS6* | 10 | 102066620 | 102066621 | NM_024747 | exon1 | c.1146_1147del | p.L383Vfs*12 | . | 0.0005 | 0.0005 | Likely pathogenic |
| *HPS6* | 10 | 102067113 | 102067114 | NM_024747 | exon1 | c.1639_1640del | p.L548Rfs*18 | . | 0.0005 | 0.0005 | Likely pathogenic |
| *HPS6* | 10 | 102067551 | 102067551 | NM_024747 | exon1 | c.C2077T | p.R693C | rs759990152 | 0.0005 | 0.0005 | Likely benign |
| *PNPLA2* | 11 | 821684 | 821684 | NM_020376 | exon3 | c.G244A | p.G82S | . | 0.0005 | 0.0005 | Likely pathogenic |
| *PNPLA2* | 11 | 824737 | 824737 | NM_020376 | exon10 | c.1390delC | p.A465Pfs*24 | . | 0.0005 | 0.0005 | Likely pathogenic |
| *CTSD* | 11 | 1753829 | 1753829 | NM_001909 | exon8 | c.C1045A | p.L349M | . | 0.0005 | 0.0005 | Uncertain significance |
| *CTSD* | 11 | 1754089 | 1754089 | NM_001909 | exon7 | c.A877G | p.I293V | rs748185051 | 0.0005 | 0.0005 | Uncertain significance |
| *CTSD* | 11 | 1757493 | 1757493 | NM_001909 | exon5 | c.T535C | p.F179L | . | 0.0005 | 0.0005 | Uncertain significance |
| *SMPD1* | 11 | 6390701 | 6390706 | NM_000543 | exon1 | c.103_108del | p.V36_L37del | rs753614903 | 0.0025 | 0.0063 | Uncertain significance |
| *SMPD1* | 11 | 6391633 | 6391640 | NM_000543 | exon2 | c.568_575del | p.P194Rfs*12 | . | 0.0035 | 0.0035 | Likely pathogenic |
| *SMPD1* | 11 | 6392060 | 6392060 | NM_000543 | exon2 | c.C995G | p.P332R | rs202081954 | 0.0055 | 0.0056 | Likely benign |
| *SMPD1* | 11 | 6394029 | 6394029 | NM_000543 | exon5 | c.G1474A | p.G492S | rs144873307 | 0.001 | 0.0033 | Likely benign |
| *SMPD1* | 11 | 6394309 | 6394309 | NM_000543 | exon6 | c.C1598T | p.P533L | rs199915216 | 0.01 | 0.0136 | Likely benign |
| *SMPD1* | 11 | 6394371 | 6394371 | NM_000543 | exon6 | c.G1660A | p.A554T | rs758811926 | 0.0005 | 0.0005 | Uncertain significance |
| *TPP1* | 11 | 6615479 | 6615479 | NM_000391 | exon10 | c.G1229A | p.G410D | . | 0.0005 | 0.0005 | Uncertain significance |
| *TPP1* | 11 | 6618817 | 6618817 | NM_000391 | exon3 | c.A188T | p.E63V | rs767113956 | 0.001 | 0.001 | Uncertain significance |
| *HPS5* | 11 | 18281986 | 18281986 | NM_181507 | exon22 | c.C3293T | p.T1098I | rs61884288 | 0.0005 | 0.0497 | Likely benign |
| *HPS5* | 11 | 18286633 | 18286633 | NM_181507 | exon19 | c.C2795T | p.A932V | . | 0.0005 | 0.0006 | Uncertain significance |
| *HPS5* | 11 | 18287646 | 18287649 | NM_181507 | exon18 | c.2603_2606del | p.I868Sfs*26 | rs753927729 | 0.0005 | 0.0005 | Likely pathogenic |
| *HPS5* | 11 | 18291531 | 18291531 | NM_181507 | exon16 | c.T2351C | p.L784P | rs769042407 | 0.0005 | 0.0012 | Uncertain significance |
| *HPS5* | 11 | 18291846 | 18291846 | NM_181507 | exon16 | c.C2036A | p.S679X | . | 0.0015 | 0.0015 | Pathogenic |
| *HPS5* | 11 | 18310896 | 18310896 | NM_181507 | exon5 | c.C322T | p.R108C | rs201474405 | 0.004 | 0.004 | Uncertain significance |
| *CTSF* | 11 | 66565923 | 66565924 | NM_003793 | exon7 | c.871_872del | p.M291Vfs*29 | rs777967311 | 0.0005 | 0.0011 | Likely pathogenic |
| *CTSF* | 11 | 66566045 | 66566045 | NM_003793 | exon6 | c.844delG | p.A282Lfs*64 | . | 0.0005 | 0.0005 | Likely pathogenic |
| *CTSF* | 11 | 66567559 | 66567559 | NM_003793 | exon3 | c.C416A | p.S139X | rs765758974 | 0.0005 | 0.0012 | Pathogenic |
| *CTSC* | 11 | 88296248 | 88296248 | NM_001814 | exon6 | c.C774A | p.C258X | . | 0.0005 | 0.0005 | Pathogenic |
| *CTSC* | 11 | 88312508 | 88312508 | NM_001814 | exon3 | c.T365C | p.M122T | . | 0.0005 | 0.0005 | Uncertain significance |
| *CTSC* | 11 | 88335079 | 88335079 | NM_001814 | exon2 | c.C176A | p.P59Q | . | 0.003 | 0.003 | Uncertain significance |
| *CTSC* | 11 | 88335082 | 88335082 | NM_001814 | exon2 | c.G173T | p.G58V | rs756512373 | 0.001 | 0.001 | Uncertain significance |
| *GNS* | 12 | 64739479 | 64739479 | NM_002076 | exon8 | c.T896C | p.V299A | rs752339367 | 0.0005 | 0.0006 | Uncertain significance |
| *GNS* | 12 | 64743280 | 64743280 | NM_002076 | exon6 | c.A653G | p.Y218C | rs777980972 | 0.0005 | 0.0005 | Likely pathogenic |
| *GNPTAB* | 12 | 101749137 | 101749137 | NM_024312 | exon20 | c.G3657T | p.L1219F | . | 0.0005 | 0.0005 | Uncertain significance |
| *GNPTAB* | 12 | 101749180 | 101749180 | NM_024312 | exon20 | c.G3614A | p.R1205Q | rs143943289 | 0.0005 | 0.0005 | Likely benign |
| *GNPTAB* | 12 | 101753376 | 101753376 | NM_024312 | exon19 | c.G3598A | p.E1200K | rs137853825 | 0.0005 | 0.0218 | Benign |
| *GNPTAB* | 12 | 101753402 | 101753402 | NM_024312 | exon19 | c.G3572A | p.R1191H | rs376398528 | 0.001 | 0.001 | Likely benign |
| *GNPTAB* | 12 | 101764513 | 101764513 | NM_024312 | exon13 | c.C2404T | p.Q802X | . | 0.0005 | 0.0005 | Pathogenic |
| *GNPTAB* | 12 | 101765009 | 101765012 | NM_024312 | exon13 | c.1905_1908del | p.E637Dfs*4 | . | 0.0005 | 0.0005 | Pathogenic |
| *GNPTAB* | 12 | 101765240 | 101765240 | NM_024312 | exon13 | c.A1677C | p.K559N | . | 0.0005 | 0.0005 | Uncertain significance |
| *GNPTAB* | 12 | 101766160 | 101766160 | NM_024312 | exon12 | c.G1543A | p.A515T | rs771144768 | 0.0005 | 0.0005 | Uncertain significance |
| *GNPTAB* | 12 | 101768083 | 101768083 | NM_024312 | exon11 | c.G1362T | p.K454N | . | 0.0005 | 0.0005 | Uncertain significance |
| *GNPTAB* | 12 | 101770414 | 101770414 | NM_024312 | exon9 | c.A1105G | p.T369A | rs779634768 | 0.0005 | 0.0005 | Uncertain significance |
| *GNPTAB* | 12 | 101770429 | 101770429 | NM_024312 | exon9 | c.C1090T | p.R364X | rs200646278 | 0.0005 | 0.0005 | Pathogenic |
| *GNPTAB* | 12 | 101786014 | 101786014 | NM_024312 | exon5 | c.A569G | p.D190G | rs34946266 | 0.0005 | 0.0005 | Uncertain significance |
| *GNPTAB* | 12 | 101786152 | 101786152 | NM_024312 | exon5 | c.C431A | p.A144D | . | 0.0005 | 0.0005 | Uncertain significance |
| *GNPTAB* | 12 | 101830599 | 101830599 | NM_024312 | exon1 | c.G77T | p.G26V | rs747570832 | 0.0005 | 0.0006 | Likely benign |
| *CLN5* | 13 | 76995215 | 76995215 | NM_006493 | exon2 | c.T326C | p.L109P | . | 0.0005 | 0.0005 | Uncertain significance |
| *GALC* | 14 | 87939915 | 87939915 | NM_000153 | exon16 | c.T1901C | p.L634S | rs138577661 | 0.0075 | 0.009 | Uncertain significance |
| *GALC* | 14 | 87939940 | 87939940 | NM_000153 | exon16 | c.G1876A | p.A626T | . | 0.0005 | 0.0005 | Uncertain significance |
| *GALC* | 14 | 87939945 | 87939945 | NM_000153 | exon16 | c.T1871A | p.V624D | . | 0.0005 | 0.0005 | Uncertain significance |
| *GALC* | 14 | 87941472 | 87941472 | NM_000153 | exon15 | c.G1757A | p.G586D | . | 0.0005 | 0.0005 | Uncertain significance |
| *GALC* | 14 | 87945607 | 87945607 | NM_000153 | exon14 | c.T1616C | p.I539T | . | 0.0005 | 0.0005 | Uncertain significance |
| *GALC* | 14 | 87945631 | 87945631 | NM_000153 | exon14 | c.G1592A | p.R531H | rs200378205 | 0.0005 | 0.0007 | Likely pathogenic |
| *GALC* | 14 | 87963436 | 87963436 | NM_000153 | exon10 | c.A1109T | p.Y370F | rs148638986 | 0.0005 | 0.0005 | Uncertain significance |
| *GALC* | 14 | 87965532 | 87965532 | NM_000153 | exon9 | c.G1006A | p.V336M | rs185073540 | 0.0005 | 0.0129 | Likely benign |
| *GALC* | 14 | 87968335 | 87968335 | NM_000153 | exon8 | c.C908T | p.S303F | rs756352952 | 0.0005 | 0.0005 | Pathogenic |
| *CLN6* | 15 | 68211315 | 68211315 | NM_017882 | exon5 | c.G490T | p.D164Y | . | 0.001 | 0.001 | Uncertain significance |
| *CLN6* | 15 | 68218576 | 68218576 | NM_017882 | exon2 | c.T158C | p.L53P | rs780955024 | 0.0005 | 0.0005 | Uncertain significance |
| *HEXA* | 15 | 72345537 | 72345537 | NM_000520 | exon13 | c.G1435A | p.A479T | rs145012038 | 0.0105 | 0.0111 | Likely benign |
| *HEXA* | 15 | 72350530 | 72350530 | NM_000520 | exon7 | c.T793C | p.S265P | . | 0.0005 | 0.0005 | Likely pathogenic |
| *HEXA* | 15 | 72350578 | 72350578 | NM_000520 | exon7 | c.C745T | p.R249W | rs138058578 | 0.0005 | 0.0012 | Uncertain significance |
| *GNPTG* | 16 | 1351970 | 1351970 | NM_032520 | exon1 | c.C5T | p.A2V | . | 0.0015 | 0.0015 | Uncertain significance |
| *GNPTG* | 16 | 1362096 | 1362096 | NM_032520 | exon6 | c.G376A | p.G126S | rs775359476 | 0.0005 | 0.0005 | Likely pathogenic |
| *GNPTG* | 16 | 1362255 | 1362255 | NM_032520 | exon7 | c.C461T | p.P154L | rs754679713 | 0.0005 | 0.0006 | Likely benign |
| *GNPTG* | 16 | 1362310 | 1362310 | NM_032520 | exon7 | c.C516A | p.H172Q | rs377440797 | 0.0005 | 0.0005 | Uncertain significance |
| *GNPTG* | 16 | 1362482 | 1362482 | NM_032520 | exon8 | c.G557A | p.R186Q | rs139997459 | 0.0005 | 0.001 | Likely benign |
| *GNPTG* | 16 | 1362526 | 1362526 | NM_032520 | exon8 | c.A601G | p.T201A | rs764889321 | 0.0005 | 0.0006 | Likely benign |
| *GNPTG* | 16 | 1362648 | 1362648 | NM_032520 | exon9 | c.C647T | p.A216V | rs777120261 | 0.001 | 0.0019 | Likely benign |
| *GNPTG* | 16 | 1363060 | 1363060 | NM_032520 | exon11 | c.887delG | p.G297Vfs*37 | . | 0.0005 | 0.0005 | Likely pathogenic |
| *CLN3* | 16 | 28487537 | 28487537 | NM_001042432 | exon7 | c.C379T | p.R127W | rs201168980 | 0.0025 | 0.0025 | Likely benign |
| *GALNS* | 16 | 88824811 | 88824811 | NM_000512 | exon11 | c.G1198A | p.A400T | rs2303270 | 0.0005 | 0.0006 | Likely pathogenic |
| *GALNS* | 16 | 88826714 | 88826714 | NM_000512 | exon10 | c.G1127A | p.R376Q | rs150734270 | 0.0035 | 0.0042 | Uncertain significance |
| *GALNS* | 16 | 88832003 | 88832003 | NM_000512 | exon9 | c.G997A | p.G333S | . | 0.0005 | 0.0005 | Likely pathogenic |
| *GALNS* | 16 | 88832047 | 88832047 | NM_000512 | exon9 | c.T953G | p.M318R | rs746756997 | 0.001 | 0.0012 | Likely pathogenic |
| *GALNS* | 16 | 88841055 | 88841055 | NM_000512 | exon4 | c.C359T | p.S120L | rs112454391 | 0.0005 | 0.0134 | Benign |
| *GALNS* | 16 | 88842781 | 88842781 | NM_000512 | exon2 | c.C169T | p.P57S | rs759564640 | 0.0005 | 0.0006 | Likely benign |
| *GALNS* | 16 | 88856761 | 88856761 | NM_000512 | exon1 | c.C117G | p.D39E | . | 0.0005 | 0.0005 | Likely pathogenic |
| *CTNS* | 17 | 3648886 | 3648886 | NM_004937 | exon5 | c.C180G | p.I60M | . | 0.0005 | 0.0005 | Uncertain significance |
| *CTNS* | 17 | 3655321 | 3655321 | NM_004937 | exon7 | c.C430T | p.P144S | rs758203278 | 0.0005 | 0.0006 | Uncertain significance |
| *CTNS* | 17 | 3656487 | 3656487 | NM_004937 | exon8 | c.T462G | p.S154R | rs77453839 | 0.0005 | 0.0006 | Uncertain significance |
| *CTNS* | 17 | 3656536 | 3656536 | NM_004937 | exon8 | c.G511A | p.V171M | rs769251750 | 0.0005 | 0.0005 | Uncertain significance |
| *CTNS* | 17 | 3656703 | 3656703 | NM_004937 | exon9 | c.G589A | p.G197R | rs113994207 | 0.001 | 0.001 | Likely pathogenic |
| *CTNS* | 17 | 3656733 | 3656733 | NM_004937 | exon9 | c.T619G | p.F207V | . | 0.0005 | 0.0005 | Uncertain significance |
| *CTNS* | 17 | 3660241 | 3660241 | NM_004937 | exon12 | c.T976C | p.W326R | rs372665052 | 0.0005 | 0.0006 | Uncertain significance |
| *NAGLU* | 17 | 42543450 | 42543450 | NM_000263 | exon6 | c.C1444T | p.R482W | rs104894596 | 0.0005 | 0.0005 | Likely pathogenic |
| *NAGLU* | 17 | 42543544 | 42543544 | NM_000263 | exon6 | c.A1538G | p.N513S | rs200465065 | 0.0005 | 0.0005 | Uncertain significance |
| *NAGLU* | 17 | 42543568 | 42543568 | NM_000263 | exon6 | c.C1562T | p.P521L | rs104894595 | 0.0005 | 0.0005 | Likely pathogenic |
| *NAGLU* | 17 | 42543699 | 42543699 | NM_000263 | exon6 | c.C1693T | p.R565W | rs104894597 | 0.0005 | 0.0006 | Likely pathogenic |
| *NAGLU* | 17 | 42543789 | 42543789 | NM_000263 | exon6 | c.G1783A | p.G595R | rs773277748 | 0.0005 | 0.0005 | Uncertain significance |
| *NAGLU* | 17 | 42544009 | 42544009 | NM_000263 | exon6 | c.2003delC | p.N669Tfs*138 | . | 0.0005 | 0.0005 | Likely pathogenic |
| *GRN* | 17 | 44349704 | 44349704 | NM_002087 | exon4 | c.G302A | p.R101Q | rs201686997 | 0.0005 | 0.0006 | Likely benign |
| *GRN* | 17 | 44350754 | 44350754 | NM_002087 | exon7 | c.G662C | p.C221S | rs758322775 | 0.0005 | 0.0025 | Uncertain significance |
| *GAA* | 17 | 80105089 | 80105089 | NM_000152 | exon2 | c.G503A | p.R168Q | rs376685205 | 0.0025 | 0.0025 | Uncertain significance |
| *GAA* | 17 | 80107569 | 80107569 | NM_000152 | exon4 | c.G705A | p.T235T | rs2304846 | 0.0005 | 0.0029 | Likely benign |
| *GAA* | 17 | 80107616 | 80107616 | NM_000152 | exon4 | c.C752T | p.S251L | rs200856561 | 0.002 | 0.0029 | Likely benign |
| *GAA* | 17 | 80107625 | 80107625 | NM_000152 | exon4 | c.C761T | p.S254L | rs577915581 | 0.002 | 0.0029 | Uncertain significance |
| *GAA* | 17 | 80108818 | 80108818 | NM_000152 | exon8 | c.T1316A | p.M439K | rs747610090 | 0.0005 | 0.0006 | Likely pathogenic |
| *GAA* | 17 | 80110014 | 80110014 | NM_000152 | exon9 | c.G1396T | p.V466F | . | 0.0005 | 0.0005 | Likely pathogenic |
| *GAA* | 17 | 80112922 | 80112922 | NM_000152 | exon14 | c.C1935A | p.D645E | rs28940868 | 0.0035 | 0.0037 | Pathogenic |
| *GAA* | 17 | 80112992 | 80112992 | NM_000152 | exon14 | c.C2005A | p.P669T | rs752507985 | 0.0005 | 0.0012 | Uncertain significance |
| *GAA* | 17 | 80113008 | 80113010 | NM_000152 | exon14 | c.2021_2023del | p.N675del | rs773958269 | 0.0005 | 0.0005 | Pathogenic |
| *GAA* | 17 | 80113309 | 80113309 | NM_000152 | exon15 | c.C2132G | p.T711R | rs759292700 | 0.0025 | 0.0025 | Uncertain significance |
| *GAA* | 17 | 80113344 | 80113344 | NM_000152 | exon15 | c.G2167A | p.V723M | rs767247397 | 0.0005 | 0.0005 | Likely pathogenic |
| *GAA* | 17 | 80116992 | 80116992 | NM_000152 | exon16 | c.G2214A | p.W738X | . | 0.0005 | 0.0005 | Pathogenic |
| *GAA* | 17 | 80117015 | 80117015 | NM_000152 | exon16 | c.G2237A | p.W746X | rs752921215 | 0.0005 | 0.0005 | Pathogenic |
| *SGSH* | 17 | 80212107 | 80212107 | NM_000199 | exon7 | c.T913G | p.W305G | . | 0.0005 | 0.0005 | Uncertain significance |
| *SGSH* | 17 | 80212269 | 80212269 | NM_000199 | exon7 | c.G751A | p.G251R | rs528633031 | 0.0005 | 0.0005 | Likely pathogenic |
| *SGSH* | 17 | 80213881 | 80213881 | NM_000199 | exon6 | c.C668T | p.P223L | rs760542684 | 0.0005 | 0.0005 | Uncertain significance |
| *SGSH* | 17 | 80214327 | 80214327 | NM_000199 | exon5 | c.C508G | p.P170A | rs527760024 | 0.0005 | 0.0006 | Uncertain significance |
| *SGSH* | 17 | 80214616 | 80214616 | NM_000199 | exon4 | c.C505T | p.R169W | rs770108057 | 0.0005 | 0.0005 | Uncertain significance |
| *SGSH* | 17 | 80214666 | 80214666 | NM_000199 | exon4 | c.T455G | p.I152S | . | 0.0005 | 0.0005 | Uncertain significance |
| *SGSH* | 17 | 80214672 | 80214672 | NM_000199 | exon4 | c.G449A | p.R150Q | rs104894638 | 0.0005 | 0.0005 | Pathogenic |
| *MAN2B1* | 19 | 12647487 | 12647487 | NM_000528 | exon22 | c.G2776A | p.D926N | . | 0.0005 | 0.0005 | Uncertain significance |
| *MAN2B1* | 19 | 12648260 | 12648260 | NM_000528 | exon21 | c.C2579T | p.A860V | rs749425508 | 0.0005 | 0.0006 | Likely benign |
| *MAN2B1* | 19 | 12649174 | 12649174 | NM_000528 | exon20 | c.G2398C | p.G800R | . | 0.0005 | 0.0005 | Likely pathogenic |
| *MAN2B1* | 19 | 12658509 | 12658509 | NM_000528 | exon8 | c.A1028C | p.Q343P | . | 0.0005 | 0.0005 | Uncertain significance |
| *MAN2B1* | 19 | 12663403 | 12663403 | NM_000528 | exon6 | c.G823A | p.D275N | . | 0.0005 | 0.0005 | Uncertain significance |
| *MAN2B1* | 19 | 12663706 | 12663706 | NM_000528 | exon5 | c.A760G | p.T254A | rs201109710 | 0.0005 | 0.0005 | Uncertain significance |
| *CTSA* | 20 | 45891702 | 45891702 | NM_000308 | exon2 | c.C134T | p.P45L | rs746311995 | 0.001 | 0.0012 | Likely benign |
| *CTSA* | 20 | 45892727 | 45892727 | NM_000308 | exon6 | c.C447T | p.V149V | rs757609079 | 0.0025 | 0.0025 | Likely benign |
| *CTSA* | 20 | 45895089 | 45895089 | NM_000308 | exon11 | c.G1044C | p.K348N | rs557995825 | 0.0005 | 0.0005 | Uncertain significance |
| *CTSA* | 20 | 45895103 | 45895103 | NM_000308 | exon11 | c.C1058T | p.P353L | rs142302144 | 0.001 | 0.001 | Likely benign |
| *CTSA* | 20 | 45897786 | 45897786 | NM_000308 | exon13 | c.G1234A | p.V412M | . | 0.0005 | 0.0005 | Uncertain significance |
| *CTSA* | 20 | 45898426 | 45898426 | NM_000308 | exon15 | c.1420dupT | p.L475Pfs*17 | . | 0.0005 | 0.0005 | Likely pathogenic |
| *HPS4* | 22 | 26453306 | 26453306 | NM_022081 | exon14 | c.2054delC | p.P685Lfs*17 | . | 0.0005 | 0.0005 | Pathogenic |
| *HPS4* | 22 | 26457896 | 26457896 | NM_022081 | exon13 | c.T1918C | p.F640L | rs760087525 | 0.0005 | 0.0005 | Uncertain significance |
| *HPS4* | 22 | 26457958 | 26457958 | NM_022081 | exon13 | c.C1856T | p.P619L | rs374238081 | 0.0005 | 0.0005 | Uncertain significance |
| *HPS4* | 22 | 26468621 | 26468621 | NM_022081 | exon8 | c.T599C | p.I200T | . | 0.0005 | 0.0005 | Uncertain significance |
| *HPS4* | 22 | 26472842 | 26472842 | NM_022081 | exon5 | c.T374C | p.L125P | . | 0.0005 | 0.0005 | Uncertain significance |
| *NAGA* | 22 | 42060977 | 42060977 | NM_000262 | exon8 | c.C1048T | p.R350C | . | 0.0005 | 0.0005 | Uncertain significance |
| *NAGA* | 22 | 42061053 | 42061053 | NM_000262 | exon8 | c.C972G | p.I324M | rs763141651 | 0.0005 | 0.0005 | Uncertain significance |
| *NAGA* | 22 | 42062867 | 42062867 | NM_000262 | exon7 | c.A917G | p.N306S | rs754396207 | 0.001 | 0.001 | Likely benign |
| *NAGA* | 22 | 42062924 | 42062924 | NM_000262 | exon7 | c.G860A | p.R287H | rs374542831 | 0.0005 | 0.0005 | Likely benign |
| *NAGA* | 22 | 42062963 | 42062963 | NM_000262 | exon7 | c.C821T | p.T274M | rs564028305 | 0.001 | 0.001 | Likely benign |
| *NAGA* | 22 | 42062976 | 42062976 | NM_000262 | exon7 | c.A808G | p.M270V | . | 0.0005 | 0.0005 | Likely benign |
| *NAGA* | 22 | 42066715 | 42066715 | NM_000262 | exon5 | c.C592A | p.P198T | rs139027070 | 0.0015 | 0.0015 | Likely benign |
| *NAGA* | 22 | 42066766 | 42066766 | NM_000262 | exon5 | c.C541T | p.R181C | rs367943149 | 0.0005 | 0.0016 | Likely benign |
| *NAGA* | 22 | 42067274 | 42067274 | NM_000262 | exon4 | c.T341G | p.L114R | . | 0.0005 | 0.0005 | Uncertain significance |
| *NAGA* | 22 | 42067775 | 42067775 | NM_000262 | exon3 | c.T314C | p.L105P | rs759251501 | 0.0005 | 0.0012 | Uncertain significance |
| *NAGA* | 22 | 42067784 | 42067784 | NM_000262 | exon3 | c.T305C | p.I102T | rs763977203 | 0.0005 | 0.0006 | Uncertain significance |
| *ARSA* | 22 | 50625330 | 50625330 | NM_000487 | exon8 | c.1344dupC | p.G449Rfs*124 | rs761555167 | 0.0005 | 0.0006 | Pathogenic |
| *ARSA* | 22 | 50625330 | 50625330 | NM_000487 | exon8 | c.G1345A | p.G449R | rs117341984 | 0.004 | 0.004 | Likely benign |
| *ARSA* | 22 | 50625378 | 50625378 | NM_000487 | exon8 | c.C1297G | p.L433V | rs201693608 | 0.004 | 0.0068 | Likely benign |
| *ARSA* | 22 | 50625953 | 50625953 | NM_000487 | exon6 | c.C1090G | p.L364V, | . | 0.0005 | 0.0005 | Uncertain significance |
| *ARSA* | 22 | 50626012 | 50626012 | NM_000487 | exon6 | c.C1031T | p.A344V | . | 0.0005 | 0.0005 | Uncertain significance |
| *ARSA* | 22 | 50626863 | 50626863 | NM_000487 | exon3 | c.C655T | p.R219C | . | 0.0005 | 0.0005 | Likely pathogenic |
| *ARSA* | 22 | 50626953 | 50626953 | NM_000487 | exon3 | c.G565A | p.V189M | rs774085931 | 0.001 | 0.001 | Likely benign |
| *ARSA* | 22 | 50627004 | 50627004 | NM_000487 | exon3 | c.G514T | p.G172C | rs74315271 | 0.0005 | 0.0005 | Uncertain significance |
| *ARSA* | 22 | 50627007 | 50627007 | NM_000487 | exon3 | c.G511A | p.D171N | rs74315466 | 0.002 | 0.0035 | Likely benign |

ACMG: American College of Medical Genetics and Genomics; MAF: maximum minor allele frequency from the Exome Aggregation Consortium (ExAC), 1000 Genomes, GenomAD, and Taiwan biobank database
